# Processing of Polyvinyl Acetate Phthalate in Hot-Melt Extrusion—Preparation of Amorphous Solid Dispersions

**DOI:** 10.3390/pharmaceutics12040337

**Published:** 2020-04-09

**Authors:** Marius Monschke, Kevin Kayser, Karl G. Wagner

**Affiliations:** Department of Pharmaceutical Technology and Biopharmaceutics, University of Bonn, 53121 Bonn, Germany; marius.monschke@uni-bonn.de (M.M.); k.kayser@uni-bonn.de (K.K.)

**Keywords:** hot-melt extrusion, amorphous solid dispersion, polyvinyl acetate phthalate, PVAP, dissolution

## Abstract

The preparation of amorphous solid dispersions (ASDs) is a suitable approach to overcome solubility-limited absorption of poorly soluble drugs. In particular, pH-dependent soluble polymers have proven to be an excellently suitable carrier material for ASDs. Polyvinyl acetate phthalate (PVAP) is a polymer with a pH-dependent solubility, which is as yet not thoroughly characterized regarding its suitability for a hot-melt extrusion process. The objective of this study was to assess the processability of PVAP within a hot-melt extrusion process with the aim of preparing an ASD. Therefore, the influence of different process parameters (temperature, feed-rate) on the degree of degradation, solid-state and dissolution time of the neat polymer was studied. Subsequently, drug-containing ASDs with indomethacin (IND) and dipyridamole (DPD) were prepared, respectively, and analyzed regarding drug content, solid-state, non-sink dissolution performance and storage stability. PVAP was extrudable in combination with 10% (w/w) PEG 3000 as plasticizer. The dissolution time of PVAP was only slightly influenced by different process parameters. For IND no degradation occurred in combination with PVAP and single phased ASDs could be generated. The dissolution performance of the IND-PVAP ASD at pH 5.5 was superior and at pH 6.8 equivalent compared to commonly used polymers hydroxypropylmethylcellulose acetate succinate (HPMCAS) and Eudragit L100-55.

## 1. Introduction

An emerging number of new chemical entities (NCE) are poorly soluble compounds, associated with a limited oral bioavailability [1,2]. Amorphous solid dispersions (ASDs) have been proven to be able to increase oral bioavailability [3,4,5,6]. While an amorphized drug tends towards recrystallization due to higher state of energy [7], ASDs may ensure physical stability by preserving the molecularly dispersed or amorphous state of the drug within a polymeric matrix [8]. Thereby, the drug can be in a thermodynamically stable state when its saturation solubility within the polymer is not exceeded at room temperature, or kinetically stabilized by means of reduced molecular mobility and increased glass transition temperature [9,10,11]. A further decisive feature of an ASD is the generation of a supersaturated solution, facilitated by an increased wettability and presence of the drug in the amorphous state [12]. Subsequently, the supersaturated state may be maintained by the precipitation inhibitory properties of the polymer [13]. 

Several polymers have been established as carriers for ASDs [14,15] and pH-dependent soluble polymers have also been shown to be particularly well suited [16,17,18,19]. These polymers are capable of protecting an acid-labile drug from the gastric juice [20], or from varying gastric pH-conditions [21]. Furthermore, they are expected to dissolve at the site of absorption, avoiding a premature release in the stomach, which could cause an early precipitation of the drug [22]. Commonly used pH-dependent soluble polymers for ASDs include hydroxypropylmethylcellulose acetate succinate (HPMCAS), hydroxypropylmethylcellulose phthalate (HPMCP) and different grades of poly(meth)acrylates (Eudragit L100-55/L100/S100/FS) [17,23,24]. 

Besides spray drying, hot-melt extrusion (HME) is a well-established production technique for ASDs [25,26]. HME can be considered as beneficial compared to spray drying when it comes to the adjustment of the particle size, since tailored downstream processing can easily be applied. Zhang et al. (2018) showed that the particle size profoundly impacted the performance of an ASD, where a moderate particle size was superior to very fine powder due to the avoidance of a premature release of the weakly basic drug. Zheng et al. (2019) observed that finer ASD particles tended towards faster recrystallization as compared to more coarse particles [27,28]. 

Despite the aforementioned merits of pH-dependent soluble polymers, they can be quite challenging within the HME process, due to a high melt viscosity, high glass transition temperature and occasionally low degradation temperature [29]. HPMCAS is widely used and is relatively easily extrudable with decent thermal stability and there is no need for a plasticizer [30]. On the contrary, HPMCP and the different Eudragit grades were revealed to be more problematic, due to a considerably low degradation onset and high melt viscosity [31]. By an addition of a suitable plasticizer, the processability of these polymers could be facilitated [32,33]. 

Polyvinyl acetate phthalate (PVAP) is another polymer with pH-dependent soluble properties, which is not yet well characterized regarding its suitability for an HME process. There is only one study available in which PVAP was examined related to HME. Mehuys et al. (2005) developed a gastro-retentive system with PVAP, but needed high amounts of plasticizer (40%, *w*/*w*) to obtain an extrudable blend, which resulted in a low storage stability [34]. Despite application of PVAP as a polymeric carrier for ASDs via rotary evaporation, spray drying, ultra-rapid freezing and fluidized bed layering [35,36,37,38], at this time there is no study available with the objective to prepare an ASD with PVAP by HME. 

**Figure 1:**
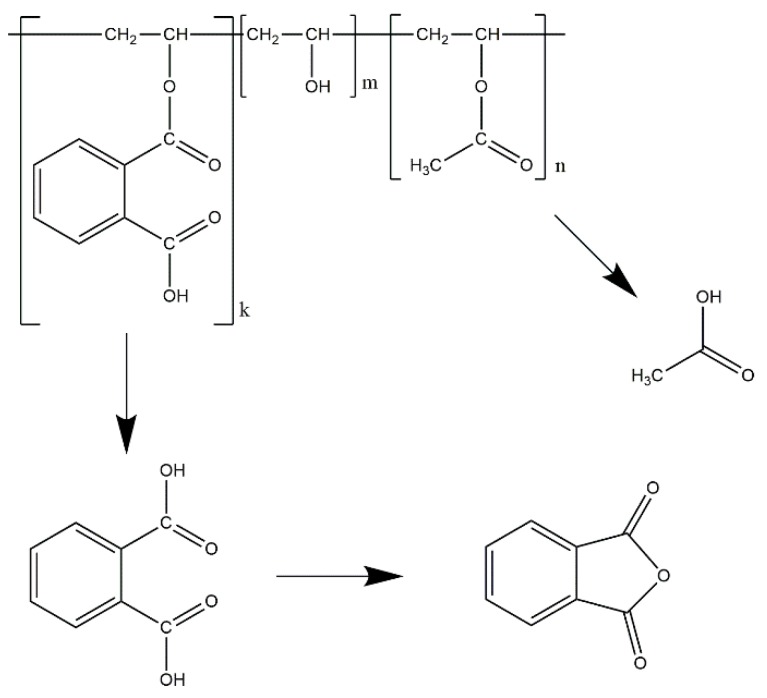
Chemical structure of polyvinyl acetate phthalate (PVAP) including a possible heat-induced degradation pathway.

The objective of this study was to assess the processability of PVAP in an HME process. Initially, a screening for a suitable plasticizer was performed in order to obtain an extrudable blend. Subsequently, this blend was processed applying different process parameters to study the influence on solid state, degree of degradation and dissolution time. The degree of degradation was investigated with respect to the content of free acids because one concern about processing PVAP via HME is a possible cleavage of the phthalate and acetate moieties (Figure 1). Finally, two poorly soluble model compounds were used to prepare drug-containing ASDs to investigate their compatibility with PVAP. The selection of the model compounds was based on their expected reactivity with PVAP and their degradation products. Dipyridamole (DPD) contains alcoholic groups which are prone to undergo heat-induced esterification [39]. Indomethacin (IND) on the contrary has no reactive groups like alcohols or primary/secondary amines and is therefore expected to stay stable. The resulting drug-containing ASDs were further evaluated for solid state including storage stability and dissolution performance. Eventually, the drug-containing PVAP ASDs were compared to the commonly used pH-dependent soluble polymers HPMCAS and Eudragit L100-55 regarding solid-state and dissolution performance. 

## 2. Materials and Methods 

### 2.1. Materials

Polyvinyl acetate phthalate (PVAP, Phthalavin) was kindly donated by Colorcon (Harleysville, PA, USA). HPMCAS LG (hydroxypropylmethylcellulose acetate succinate) was provided by Shin-Etsu Chemical (Tokyo, Japan). Eudragit L100-55 (methacrylic acid ethylacrylate copolymer, ratio 1:1, L100-55) was provided by Evonik (Darmstadt, Germany). The model drugs indomethacin and dipyridamole were obtained from Swapnroop Drugs and Pharmaceuticals (Aurangabad, India). Particle sizes of the active ingredients are given in Appendix B (determined by laser diffraction). PEG 3000, triethyl citrate (TEC) and methylparabene were purchased from Alfa Aesar (Haverhill, MA, USA). Citric acid was purchased from Carl Roth (Karlsruhe, Germany) and sorbitol was provided by Merck (Darmstadt, Germany). 

### 2.2. Thermogravimetric Analysis (TGA)

In order to investigate the thermal stability of PVAP, the percentage weight loss was determined by using a temperature gradient from 25 to 350 °C with a heating rate of 10 K/min with a nitrogen purge of 20 mL/min (Perkin-Elmer Thermogravimetric Analyzer TGA 7, Waltham, MA, USA). Additionally, an isocratic investigation for 30 min was performed at 120 and 140 °C, respectively. All experiments were performed in platinum crucibles.

### 2.3. Differential Scanning Calorimetry (DSC)

For DSC measurements a Mettler-Toledo DSC 2 equipped with a nitrogen cooling system was used (Mettler-Toledo, Gießen, Germany). Accurately weighed samples of approximately 10 mg were placed in aluminum pans with a pierced lid. A multi-frequency temperature modulation (TOPEM-mode) with an underlying heating rate of 2 K/min with a constant nitrogen-purge (30 mL/min) was used to determine glass transition temperatures of raw materials and formulations. 

### 2.4. X-Ray Powder Diffraction (XRPD)

X-ray powder diffraction experiments were carried out on an X’Pert MRD Pro (PANalytical, Almelo, the Netherlands) in reflection mode with an X’Celerator detector and nickel filtered CuKa1 radiation at 45 kV and 40 mA. The scanning range was set from 4° to 45° 2θ with a step size of 0.017° 2θ.

### 2.5. Screening for Plasticizers

From preliminary experiments, it is known that PVAP alone cannot be processed in a hot-melt extrusion process due to its high melt viscosity, leading to a motor overload. In order to gain an extrudable blend a screening for suitable plasticizers was performed, where five common plasticizers were investigated (triethyl citrate, methylparabene, sorbitol, citric acid and PEG 3000). Therefore, 500 mg of a mixture of PVAP and plasticizer in a ratio of 90:10 was dissolved in 5 mL of a 1:1 mixture of methylene chloride/methanol. The solution was placed into a petri dish and dried at 50 °C. Subsequently, a thermal analysis was performed according to Section 2.3. in order to determine glass transition temperatures and possible melting endotherms. 

### 2.6. Melt Rheology 

Small amplitude oscillatory shear (SAOS) rheology was performed using a Haake Mars III (Thermo Scientific, Karlsruhe, Germany) equipped with a 8-mm plate-plate geometry. Frequency sweeps were carried out from 10 to 0.1 Hz at various temperatures at a constant amplitude of 0.5%. The amplitude was within the linear viscoelastic range, which was verified by amplitude sweeps. 

### 2.7. Hot-Melt Extrusion (HME) 

Hot-melt extrusion was performed with a Three-Tec 12 mm co-rotating twin-screw extruder ZE 12 (Three-Tec, Seon, Switzerland) with a functional length of 25:1 L/D equipped with a 2-mm die. Prior blending and extrusion all substances were sieved through a 125-µm mesh. All mixtures for extrusion were blended for 10 min using a turbula mixer (Willy A. Bachofen AG Maschinenfabrik, Switzerland. The plasticized PVAP blend was extruded at two different temperatures (120 and 140 °C) and additionally with three different feed-rates (1, 2, 3 g/min) to investigate the influence of the temperature and the mean residence time on the content of free acids, solid-state and dissolution time. The drug containing ASDs exhibited a drug load of 10% indomethacin or dipyridamole and 10% plasticizer (PEG 3000). They were processed at a constant temperature of 120 °C with the three feed-rates (1, 2, 3 g/min). The screw speed was set to 100 rpm and was constant for all experiments. All extrudates were milled using a mixer mill 400 MM (Retsch, Haan, Germany) and sieved through a 355 µm sieve to remove large particles. The mean residence time (MRT) was measured by the aid of red iron oxide and calculated using ExtruVis3 (ExtruVis, Riedstadt, Germany). The specific mechanical energy (SME) was calculated according to Equation (1):

(1) $$SME=\frac{2\times \pi \times n\times \tau }{60\times \dot{m}}$$

where *n* is the screw speed, *τ* is the torque and *ṁ* is the throughput.

### 2.8. High Performance Liquid Chromatography (HPLC)

HPLC experiments were conducted on a Waters 2696 separation module coupled with a Waters 996 photodiode array detector (Waters, Milford, MA, USA). The column was an Inertsil RP-18 column (250 × 4.6 mm, 5 µm, GL Sciences, Tokyo, Japan) operated at 25 °C with a constant flow of 1 mL/min. For the quantification of phthalic acid, phthalic anhydride and indomethacin the mobile phase consisted of 20% aqueous trifluoroacetic acid (0.1%), 40% methanol and 40% acetonitrile. For each component, a calibration curve was recorded at 210 nm in a range of 10 to 500 µg/mL, 10 to 230 µg/mL and 20 to 210 µg/µL, respectively. Then, 50 mg of placebo extrudate or IND ASD were accurately weighed and dissolved in 50.0 mL methanol, and 10 µL of the solution were injected and the contents were evaluated by aid of the calibration curves. Dipyridamole was quantified using a mobile phase consisting of 20% PBS (10 µM) pH 6.8, 40% methanol and 40% acetonitrile. A calibration was performed at 292 nm in a range between 20 and 200 µg/mL. Then, 50 mg of DPD ASDs were dissolved in 50.0 mL methanol and 10 µL were injected for the quantification. 

The determination of acetic acid was conducted with a LiChrospher 100 RP-18 column (125 × 4.6 mm, 5 µm, Merck Millipore, Billerica, MA, USA) with a mobile phase of 80% water (0.1% phosphoric acid) and 20% methanol. A calibration was performed at 210 nm in a range of 50 to 200 µg/mL. In order to evaluate the content of free acetic acid within the extrudates, 100 mg were dissolved in 2.5 mL THF. The solution was filled up to 50.0 mL with water to precipitate the polymer. Subsequently, the suspension was centrifuged at 15000 rpm for 15 min and the supernatant was filtered through 0.22 µm PES syringe filter (2 mL were discarded). Then, 50 µL of the obtained solution were injected into the HPLC system and the content of acetic acid was determined using the calibration curve.

### 2.9. Dissolution Time

The six differently processed placebo extrudates were studied regarding their dissolution times to investigate the influence of process conditions on the dissolution behavior of the drug-free extrudates. Samples were sieved through a 355-µm sieve and 25 mg PVAP were placed in the MiniDissolution apparatus [40] containing 20 mL buffer with a paddle speed of 75 rpm and 37 °C medium temperature for 30 min. The experiments were conducted at pH 5.5 and 6.8, respectively (McIlvaine buffer). The concentration over time of the UV-sensitive PVAP was measured online with a diode array UV/VIS spectrophotometer (Agilent 8453 Agilent Technologies GmH, Waldbronn, Germany).

### 2.10. Non-Sink Dissolution

Non-sink dissolution experiments were conducted in order to evaluate supersaturation performance of the drug-containing ASDs using the MiniDissolution apparatus with 20 mL buffer at 37 °C with a paddle speed of 75 rpm. Again, dissolution studies were performed at pH 5.5 or 6.8 for 3 h in total. ASDs equivalent to 4 mg IND or pure IND (equals a theoretical concentration of 0.2 mg/mL) were placed in each vessel. Concentration over time was measured according to dissolution time experiments. The saturation solubilities of IND at pH 5.5 and pH 6.8 were determined using the shake-flask method (Appendix A)

### 2.11. Stability Studies

The physical stability of the ASDs regarding recrystallization and dissolution performance was investigated. ASD samples in screw cap vials were stored at 25 °C and 60 % relative humidity (RH) for 6 months and under accelerated conditions of 40 °C and 75% RH for 1 and 4 weeks, respectively. The samples were analyzed for solid-state by XRPD and DSC and for dissolution performance according to Section 2.10. 

## 3. Results and Discussion

### 3.1. Thermal Analysis of Neat PVAP

**Figure 2:**
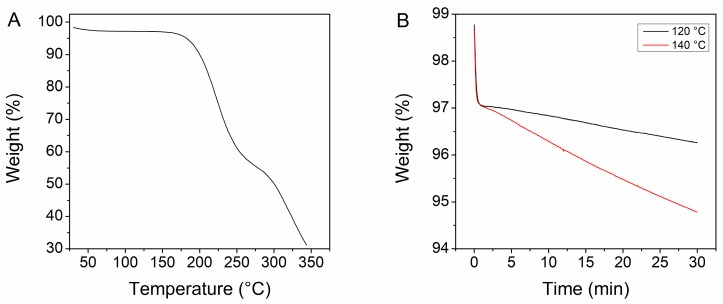
Thermogravimetric analysis of neat PVAP, (**A**) temperature gradient from 25 to 350 °C, (**B**) isothermal at 120 and 140 °C.

The glass transition temperature of neat PVAP was determined via DSC by applying a heat-cool-heat cycle to remove the thermal history of the raw material and it was found to be at 115 °C (please see supplementary data (Figure S1)). The thermal stability was initially considered in a TGA temperature gradient measurement from 25 to 350 °C (Figure 2A). The first step of weight loss was related to adsorbed moisture, which was followed by a plateau indicating the stable region. The onset of degradation (0.1% weight loss) was recorded at 145 °C. Additionally, two isothermal experiments at 120 and 140 °C were carried out to evaluate the thermal behavior at temperatures relevant for hot-melt extrusion (Figure 2B). For the experiments at 120 and 140 °C, slopes of 0.0265 and 0.0893 %/min could be observed. If these slopes are extrapolated to 10 min, which would be equivalent to a rather long MRT in an HME process, a weight loss of 0.2% and 0.8% would be obtained, respectively. This extent of weight loss can be considered as acceptable, especially if it is taken into account that usual MRTs are often significantly shorter than 10 min. However, these results only consider degradation products that are volatile, which means that non-volatile species will not be detected. Therefore, all relevant degradation products were determined by HPLC (see Section 3.3.2). 

### 3.2. Screening for Plasticizers

Since PVAP alone turned out to be unextrudable due to motor overload caused by very high melt viscosity, it was necessary to find suitable plasticizers. All PVAP/plasticizer blends (triethyl citrate, methylparabene, sorbitol, citric acid and PEG 3000) on a plasticizer level of 10% (*w*/*w*) exhibited a single glass transition temperature (Figure S2, Table S1). No melting endotherms could be observed, which indicated a single-phase system where PVAP and each plasticizer were miscible with each other. For the further course of the study, PEG 3000 was selected as plasticizer due to its ease of handling. The glass transition temperature of the PVAP/PEG 3000 blend was found to be 65.7 ± 0.1 °C.

**Figure 3:**
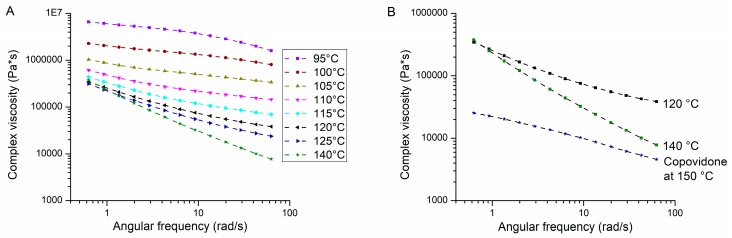
Effect of angular frequency on complex viscosity at different temperatures of the PVAP/PEG 3000 blend (**A**). Comparison of frequency sweeps of the PVAP/PEG 3000 blend at 120 and 140 °C with copovidone at 150 °C (**B**).

The selected polymer plasticizer blend was studied regarding its rheological properties. Melt rheology plays an important role for an HME process as it determines general extrudability and is also a key factor for the specific mechanical energy. The PVAP/PEG 3000 mixture was characterized by frequency sweeps at different temperatures to study the influence of shear rate on complex viscosity. As can be seen in Figure 3A, complex viscosity decreased with an increase in angular frequency. This shear-thinning behavior is common for polymeric melts due to an alignment of the polymer chains. [41,42]. Further, it could be observed that an increase in temperature led to a decrease in complex viscosity. In order to classify these results, they were compared to the well-characterized and well extrudable polymer copovidone. Figure 3B shows the frequency sweeps of PVAP/PEG 3000 at temperatures of 120 and 140 °C, which are relevant for HME and for copovidone at 150 °C (recommended standard temperature for HME). Copovidone also exhibited shear-thinning behavior, but the complex viscosity was in a distinctly lower range (25,000 to 4500 Pa·s). The complex viscosity for PVAP/PEG 3000 at 120 °C was almost parallelly shifted upwards by a power of ten (340,000 to 38,000 Pa·s). At 140 °C, the complex viscosity at low angular frequency was comparable to the experiment at 120 °C, but with increasing shear the drop in complex viscosity was more pronounced, indicating stronger shear-thinning behavior at 140 °C (375,000 to 7700 Pa·s). Since significantly higher shear-rates predominate in an HME process, no direct correlations can be drawn from the rheological measurements for the HME process. However, by comparative evaluation, it is possible to estimate the extrudability. The comparison of the melt viscosity of copovidone with the PVAP blends indicated that processing of the plasticized PVAP would be quite challenging, where relatively high torque values would be expected. In this context, Sarode et al. (2014) showed that even materials exhibiting complex viscosities of up to 1,000,000 Pa·s were extrudable [30]. 

### 3.3. Characterization of Placebo Extrudates

#### 3.3.1. Hot-Melt Extrusion and Mean Residence Time

**Figure 4:**
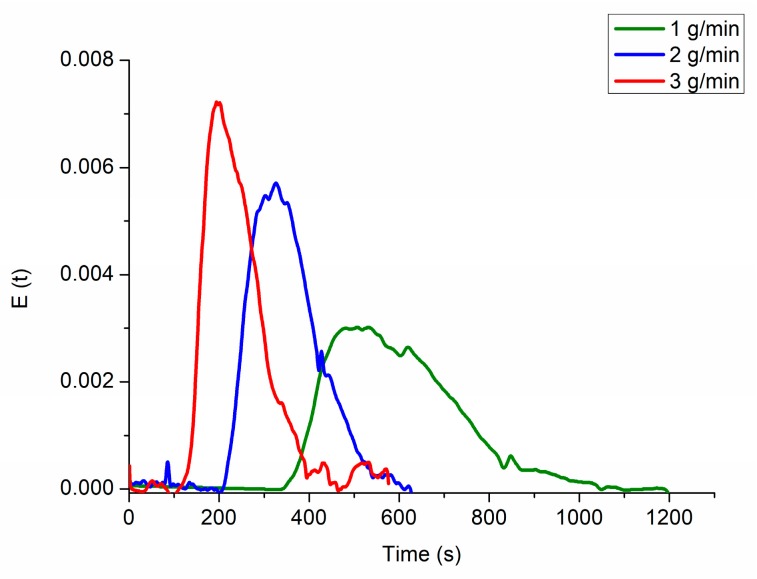
Residence time distributions at feed-rates of 1, 2 and 3 g/min.

The PVAP/PEG 3000 blend (90:10) was extruded at 120 and 140 °C with three different feed-rates, respectively (Table 1). In order to receive information about the residence time distribution of the material within the extrusion barrel in dependence of feed-rate, MRT measurements were performed. Figure 4 shows the residence time distributions for three different feed-rates. The MRT for a feed-rate of 1 g/min was found at 596 s with a comparably broad distribution. MRTs for 2 and 3 g/min were found at 351 and 238 s, respectively, whereby higher feed-rates resulted in more narrow residence time distributions. The MRT can be a valuable parameter to estimate the thermal stress that acts upon the material, which in turn can be correlated to the degree of degradation (Section 3.3.2). 

As shown in Table 1, with increasing feed-rate an increasing torque could be observed at process temperature of 120 °C, which was attributed to the higher degree of fill. At 140 °C, with increased feed-rate there was no pronounced change in torque, which was likely originated by stronger shear-thinning behavior at 140 °C. Derived from the torque as well as the throughput and the screw speed, it is possible to calculate the SME, which describes the mechanical energy put into the extruded material. The SME is another valuable parameter, which can be correlated to the degree of degradation. Table 1 depicts that the SME decreased with higher feed-rates at one specific temperature and the higher level of temperature also led to lower SMEs at the same feed-rates, attributed to lower melt viscosity at elevated temperature.

#### 3.3.2. Influence of Process Parameters on Content of Free Acids

**Figure 5:**
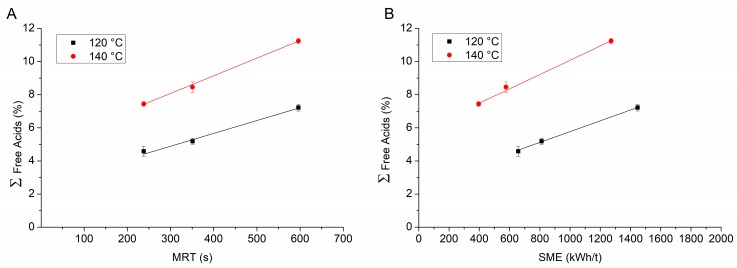
Plot of the sum of free acids against mean residence time (MRT) (**A**) and specific mechanical energy (SME) (**B**).

Since PVAP is prone to cleavage of its phthalic acid (PA) and acetic acid (AA) moieties under heat stress, the content of free acids including phthalic anhydride (PAH) (resulting from self-esterification of phthalic acid) was determined for all extrusion parameters. As Table 1 shows, the initial content of PA and AA in unprocessed PVAP was 0.70% and 0.66%, respectively. The extruded material at 120 °C exhibited a five-fold increase in PA content at the highest feed-rate and also PAH was detected in small concentrations. Only a slight increase to 0.77% AA could be detected at these process parameters. All contents of free acids increased with decreasing feed-rate at 120 °C. At 140 °C, the increase in PA compared to the as-received material was more pronounced (8.7-fold) at the highest feed-rate. Also, a higher amount of PAH could be detected compared to 120 °C (0.42% vs. 0.16%). Again, the increase in AA was not very pronounced compared to the as-received material (0.93% vs. 0.66%). Similar to the material processed at 120 °C, at 140 °C all contents of free acids increased with decreasing feed-rate. In order to assess that observed trend, the MRT as well as the SME were plotted against the sum of free acids for both temperatures. MRT and SME are more specific process parameters as they describe the duration within the extrusion barrel as well as the mechanical energy input, whereas the feed-rate does not exhibit any explanatory power. Plotting the MRT against the sum of the free acids at 120 and 140 °C resulted in a linear relationship (R^2^ = 0.9918; 0.9974) with slopes of 0.0075 and 0.0107 %/s, respectively. As compared to the results from the TGA experiments, these results suggest a more pronounced extent of degradation, which is related to the fact that TGA only detects volatile degradation products. Therefore, TGA experiments can be indicative for degradation but should be considered with care. The plot of the SME vs. the sum of the free acids also resulted in linear relationships for 120 and 140 °C (R^2^ = 0.9986; 0.9955) with slopes of 0.0033 and 0.0042 %t/kWh, respectively (Figure 5). 

As anticipated by TGA analysis, the degree of degradation at 140 °C was greater than at 120 °C and the degree of degradation could be linearly correlated to the process parameters MRT and SME. 

These results should be taken into account when designing the process window for the extrusion of PVAP. An extrusion temperature of 120 °C should rather not be exceeded if possible. A further reduction in temperature could be facilitated by the addition of higher amounts of plasticizer, whereby the physical stability should be considered. Additionally, a relatively high feed-rate would also be reasonable to limit the degradation of the polymer. The narrow process window of PVAP is a considerable limitation compared to other commonly used polymers, which often can be extruded at higher temperatures. However, if 120 °C is sufficient for the process of interest, PVAP can be an alternative carrier for ASDs. 

#### 3.3.3. Solid-State

The solid-state of the placebo extrudates was investigated for every applied process condition using XRPD and DSC. All placebo extrudates were of amorphous nature with very similar reflection patterns. Also, all glass transitions temperatures were in a range of 65 ± 3 °C, indicating no pronounced effect of process conditions on the solid-state properties. The DSC thermograms and the XRPD diffractograms can be found in the supplementary data in Figure S3.

#### 3.3.4. Dissolution Time

**Figure 6:**
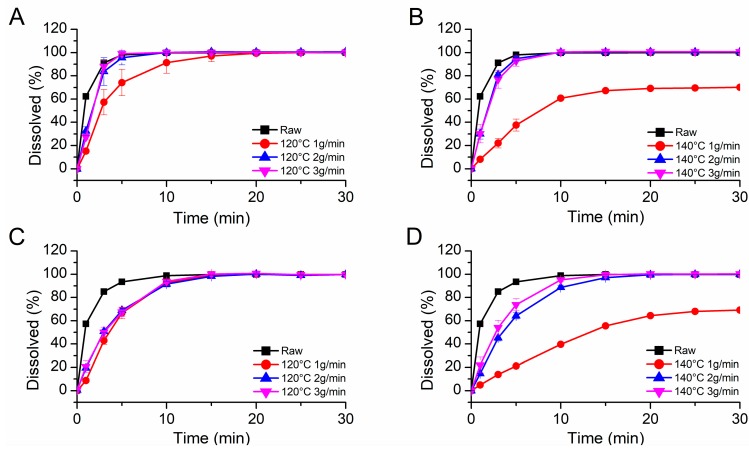
Dissolution times of the placebo extrudates at pH 6.8 (**A**,**B**) and at pH 5.5 (**C**,**D**).

In a further step, the dissolution time of powdered placebo extrudates (<355 µm) was studied for all process parameters at pH 5.5 and 6.8, respectively. Figure 6A shows the results for the placebo extrudates manufactured at 120 °C as well as for the as received material at pH 6.8. The unprocessed PVAP dissolved completely after 7.5 min at pH 6.8 and after 10 min at pH 5.5 (Figure 6C). This difference in dissolution time can be explained by the degree of ionization at the respective pH value. At pH 6.8, a higher degree of ionization is expected as compared to pH 5.5, since more acidic moieties are expected to be deprotonated, leading to a higher dissolution time. The placebo extrudate produced at 120 °C at the lowest feed-rate (1 g/min) needed 20 min at pH 6.8 and 15 min at pH 5.5 to dissolve completely. The relatively slower dissolution time compared to the unprocessed neat material was attributed to the fact that less acids were bound at the PVAP backbone for the processed material, which in turn led to a lower degree of ionization. For the highest feed-rate (3 g/min) at 120 °C, there was no difference in dissolution time at pH 6.8 compared to the neat PVAP and only a minor difference (10 vs. 15 min) at pH 5.5. The dissolution times of the placebo extrudate processed at 140 °C were only slightly different at 2 and 3 g/min compared to 120 °C. For the lowest feed-rate (1 g/min) at 140 °C, an incomplete dissolution could be observed, where only 60% was approached (Figure 6B,D), possibly due to onset of cross-linking at these conditions. Consequently, theses process conditions should be considered as insufficient. All dissolution times are summarized in Table 2.

### 3.4. Characterization of Drug-Containing ASDs

For the preparation of the drug-containing ASDs with indomethacin and dipyridamole, respectively, it was decided to apply only 120 °C for HME, as the degree of degradation was the least at this temperature. However, three feed-rates were applied again to investigate the influence of the MRT on the drug content. Both ASDs also contained 10% PEG 3000. 

#### 3.4.1. Drug Content

As mentioned above, both drugs, indomethacin and dipyridamole, were selected due to their expected compatibility with the PVAP polymer. As DPD exhibits four alcoholic groups, it was expected to be highly reactive with PA and PAH, which can be split off the PVAP backbone under heat stress. Subsequently, the free acids and the alcoholic groups of DPD can undergo an esterification. On the contrary, IND exhibits no chemical groups, which are expected to react with the free acids. 

Table 3 shows the HME process parameters and the drug content, for the IND and DPD ASDs, respectively. At the lowest feed-rate DPD degraded almost completely (99.15%). With higher feed rates (2 g/min and 3 g/min) which corresponds to a lower residence time, the degree of degradation slightly decreased (95.7% and 92.5%). However, as expected DPD can be considered as incompatible with PVAP under heat stress and due to high degree of degradation no further characterization was performed for the DPD ASDs.

As shown in Table 3, there was no significant degradation detectable for IND for all three feed-rates. This finding proved the compatibility of IND with the PVAP polymer in an HME process at 120 °C. Subsequently, for performance evaluation of PVAP as carrier for ASDs, the IND ASD was selected. 

#### 3.4.2. Solid-State

**Figure 7:**
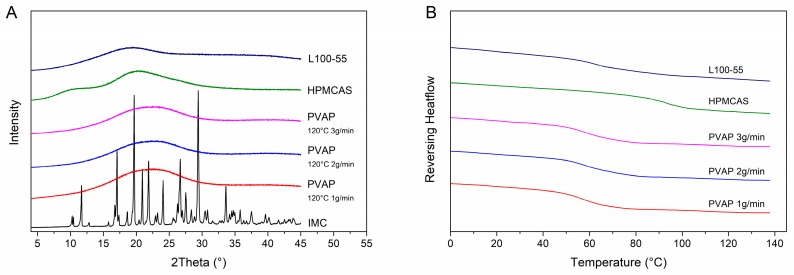
Solid-state of the drug-containing ASDs. X-ray powder diffraction (XRPD) diffractograms (**A**). Differential scanning calorimetry (DSC) thermograms (**B**).

In order to investigate the solid-state of the IND ASDs, XRPD and DSC studies were performed. As apparent in Figure 7A, the IND-PVAP ASDs were completely amorphous for all three feed-rates as no reflection peaks were visible. Also, single glass transition temperatures could be detected without differences among the three feed-rates (58.5 ± 0.5 °C), and no melting endotherms were present either (Figure 7B). 

Two further pH-dependent soluble polymers commonly used as carriers for ASDs were used, namely HPMCAS and Eudragit L100-55. The purpose was a comparative assessment of the properties and performance of the PVAP ASD with two further commonly used polymeric carriers. For both, the HPMCAS ASD and for the L100-55 ASD, completely amorphous systems were obtained. The glass transition temperature for the HPMCAS formulation was found at 92.3 ± 0.8 °C and for the L100-55 formulation at 66.1 ± 0.7 °C. 

#### 3.4.3. Non-Sink Dissolution

**Figure 8:**
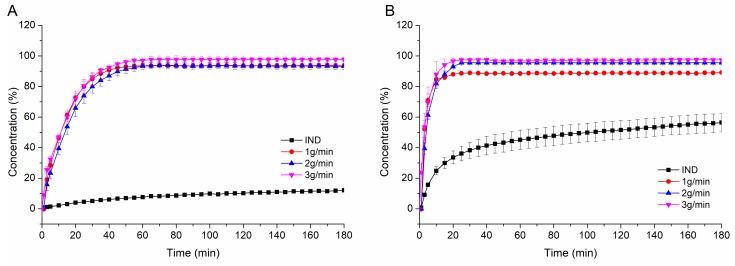
Non-sink dissolution of indomethacin (IND)-PVAP ASDs for different hot-melt extrusion (HME) process conditions at pH 5.5 (**A**) and pH 6.8 (**B**).

Figure 8 depicts the non-sink dissolution results of neat indomethacin and the IND-PVAP ASDs produced at three feed-rates at pH 5.5 (Figure 8A) and pH 6.8 (Figure 8B). At pH 5.5, all three ASDs dissolved completely within 45 min without differences among different feed-rates, generating an eight-fold supersaturated concentration compared to neat indomethacin, which was stable throughout the whole experiment. At pH 6.8, the ASDs again behaved very similar among the different feed-rates, the maximum concentration was reached after 25 min, which was twice as high as compared to neat IND. The more pronounced dissolution rate of neat IND at pH 6.8 compared to pH 5.5 was attributed to the pK_A_ of 4.5 [43]. At pH 6.8, the degree of ionization was higher as compared to pH 5.5, leading to differences in saturation solubility. The differences between the IND extrudates regarding the release rate at different pH values can be derived from the dissolution time experiments of the placebo extrudates. Dissolution time experiments revealed that PVAP dissolved faster at pH 6.8 due to degree of ionization, which could also be observed for the drug-containing ASDs. However, the placebo extrudates dissolved relatively faster than the IND ASDs at a respective pH. This is quite plausible due to the fact that the presence of a drug with a considerably high logP value (4.3) within the polymeric carrier increases the lipophilicity of the drug-containing ASD compared to the pure polymer [27,28]. Consequently, the increased lipophilicity led to a slower dissolution rate for the drug-containing ASD. Owing to that phenomenon, the minor differences in dissolution time of the placebo extrudates were levelled when it comes to dissolution of the drug-containing IND ASD.

**Figure 9:**
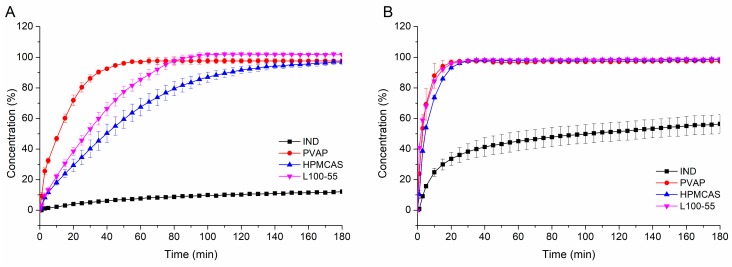
Non-sink dissolution of the IND-PVAP, - hydroxypropylmethylcellulose acetate succinate (HPMCAS), -L100-55 ASDs at pH 5.5 (**A**) and pH 6.8 (**B**).

In addition, the IND-PVAP ASD produced at 3 g/min was compared to two ASDs prepared with commonly used pH-dependent soluble polymers HPMCAS and Eudragit L100-55 regarding dissolution performance at pH 5.5 and pH 6.8, respectively. Both ASDs were prepared with a drug load of 10%, as well. As visible in Figure 9A, at pH 5.5 the PVAP ASD dissolved the fastest (within 45 min), the L100-55 formulation needed twice the time to dissolve completely (90 min) and the HPMCAS ASD even required 160 min to approach complete dissolution. In all cases the maximum concentration (level of supersaturation) was maintained throughout the whole experiment, indicating a sufficient precipitation inhibitory effect for all three polymers. At pH 6.8 (Figure 9B), all three ASDs dissolved quickly to a full extent within 25 min and this supersaturated state was again maintained over the entire period. 

The markedly faster dissolution of the PVAP ASD at pH 5.5 can be thoroughly beneficial. The small intestine represents the main site of absorption and previous studies revealed that the intestinal pH can be highly variable [44]. Vertzoni et al. (2019) pointed out that digestion leads to a high pH variability (from pH 4.8 to 6.5), which lasts for several hours [45]. Interestingly, an intake of a glass of water also causes a high pH variability in the intestine, which is accompanied by a decreased pH value. Considering that an administration of a drug product takes place by aid of a glass of water, it should be taken into account that the intestinal pH can deviate from the expected value of 6.8. Therefore, it was decided to perform dissolution experiments also at a lower pH (5.5). The dissolution results at pH 5.5 exposed a considerable advantage of PVAP towards L100-55 and HPMCAS as PVAP exhibited a markedly higher dissolution rate at that pH. For the case that the intestinal pH is around 5.5, it can be assumed that the PVAP ASD performs better and faster. For the case of “normal” pH conditions in the intestine (around pH 6.8), similar performances of the three polymeric ASDs can be expected. 

#### 3.4.4. Stability

**Figure 10:**
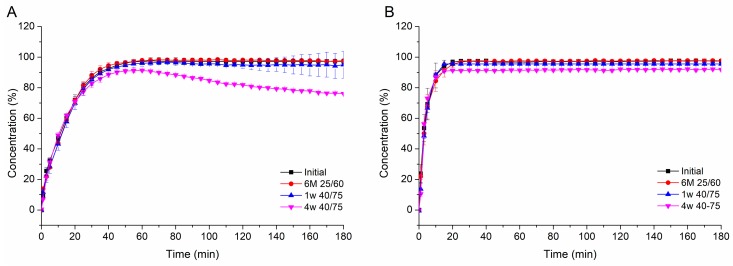
Non-sink dissolution of IND-PVAP ASDs upon storage at different conditions at pH 5.5 (**A**) and pH 6.8 (**B**).

The storage stability of the ASDs was tested at 25 °C/60% RH for six months and at accelerated conditions (40 °C/75% RH) for 1 week and 4 weeks, respectively. For the PVAP ASDs stored for six months at 25 °C/60% RH and for 1 week at 40 °C/75% RH, no differences in dissolution performance compared to the initial sample could be observed at pH 5.5 (Figure 10A), indicating a sufficient stability at these conditions. Also, no changes in solid-state (glass transition and amorphous state) were detectable (Figures S4, S5). However, the dissolution result at pH 5.5 after storage at 40 °C/75% RH for 4 weeks deviated from the other samples. Initially, it dissolved similar to the other samples, but after 60 min a slight precipitation was induced, which was accompanied with a concentration reduction from 100% to 80% within 2 h after the induction. Even though the solid-state analysis of this sample did not reveal any recrystallization (Figure S4), a shift in glass transition temperature from 58.5 ± 0.5 (initial) to 52.5 ± 1.1 °C could be detected (Figure S5). That shift might be caused by moisture adsorption, which possibly led to an undetectable phase separation of IND and PVAP. This situation might have led to an incongruent release of drug and polymer, causing an insufficient precipitation inhibition. However, at pH 6.8, all differently stored samples performed as good as the initial sample. 

For the HPMCAS and L100-55 based ASDs, no difference in dissolution performance occurred, irrespective of the storage conditions (data not shown). Also, the solid-state analysis of these ASDs revealed no changes upon storage (Figures S4, S5).

## 4. Conclusions

PVAP turned out to be processable by HME if a sufficient plasticizer (PEG 3000) was added in a reasonable amount (10%). During the HME process a cleavage of the acids PA, PAH and AA from the PVAP backbone occurred, whereby the extent cleavage was dependent on the MRT, SME and the temperature. In order to keep the degree of degradation in an acceptable range, 120 °C might not be exceeded during HME. In addition, higher feed-rates are more reasonable to ensure a relatively short MRT. Consequently, the processing window of PVAP in an HME can be considered as quite narrow. However, the influence of the cleaved acids from the PVAP backbone had only a minor impact on the dissolution time of the placebo extrudates and no significant impact on the dissolution of the drug-containing ASDs. The selection of the API for the formulation of an ASD comprising PVAP should be made with care as some chemical groups like alcohols and primary/secondary amines are prone to undergo esterification reactions with the free acids from the PVAP. Dipyridamole turned out to be incompatible with PVAP. Due to presence of alcohol groups, an almost complete degradation of dipyridamole occurred. In contrast, indomethacin proved to be compatible with PVAP within an HME process and no degradation of IND took place. The dissolution of the IND-PVAP ASD generated a stable supersaturation compared to neat IND. It exhibited a markedly faster dissolution rate at pH 5.5 compared to ASDs comprising of HPMCAS or L100-55. At pH 6.8, no difference between the different polymeric ASDs occurred. Regarding storage stability of the IND-PVAP ASD, it could be recommended to store it at or below 25 °C, because upon accelerated storage conditions a drop in performance could possibly emerge. 

## Figures and Tables

**Table 1:** Hot-melt extrusion process conditions for the placebo extrudates and contents of free acids upon extrusion.

HME Conditions				Free Acids (%)			
Temperature (°C)	Feed Rate (g/min)	Torque (%)	SME (kWh/t)	PA	PAH	AA	Sum
Physical blend	-	-	-	0.70 ± 0.02	0	0.66 ± 0.02	1.36
120	1	63.3	1447.1	5.67 ± 0.16	0.49 ± 0.04	1.06 ± 0.01	7.22
120	2	70.0	811.2	4.04 ± 0.15	0.16 ± 0.01	0.99 ± 0.03	5.19
120	3	83.3	656.8	3.65 ± 0.26	0.16 ± 0.01	0.77 ± 0.05	4.58
140	1	56.6	1271.7	9.18 ± 0.11	0.98 ± 0.02	1.07 ± 0.01	11.23
140	2	52.0	575.7	6.70 ± 0.12	0.75 ± 0.17	1.01 ± 0.02	8.46
140	3	53.4	396.0	6.09 ± 0.08	0.42 ± 0.02	0.93 ± 0.03	7.44

**Table 2:** Dissolution times of the placebo extrudates at pH 5.5 and pH 6.8, respectively.

Process Condition	pH 6.8	pH 5.5
	Dissolution Time (min)	Dissolution Time (min)
Raw material	7.5	10
120 °C, 1 g/min	20	15
120 °C, 2 g/min	10	15
120 °C, 3 g/min	5	15
140 °C, 1 g/min	incomplete	incomplete
140 °C, 2 g/min	10	20
140 °C, 3 g/min	10	15

**Table 3:** Hot-melt extrusion process parameters for the drug-containing amorphous solid dispersions (ASDs) including drug content.

Composition	HME Conditions			Content (Assay) **(%)**
	Temperature (°C)	Feed Rate (%)	Torque (%)	
IND/PEG3000/PVAP 10/10/80	120	1	49.3	99.5 ± 3.21
	120	2	60.0	99.3 ± 1.13
	120	3	66.7	104.2 ± 0.04
DPD/PEG3000/PVAP 10/10/80	120	1	50.0	0.85 ± 0.01
	120	2	56.6	4.3 ± 0.01
	120	3	66.7	7.5 ± 0.12

## Supplementary Materials

The following are available online at https://www.mdpi.com/1999-4923/12/4/337/s1, Figure S1: DSC thermogram of neat PVAP, Figure S2: DSC thermograms of plasticized PVAP films, Figure S3: (A) XRPD diffractograms and (B) DSC thermograms of PVAP/PEG 3000 (90/10) mixtures extruded at various process parameters, Figure S4: XRPD diffractograms of PVAP, HPMCAS and Eudragit L100-55 ASDs upon storage under different conditions, Figure S5: DSC thermograms of PVAP, HPMCAS, Eudragit L100-55 ASDs upon storage under different conditions, Table S1: Compositions and glass transition temperatures of plasticized PVAP films.

## Appendix A

**Table A1:** Saturation solubility of indomethacin at pH 5.5 and pH 6.8.

Medium	Saturation Solubility (µg/mL)
pH 5.5	42.6 ± 0.8
pH 6.8	388.5 ± 14.5

## Appendix B

**Table A2:** Particle sizes of indomethacin and dipyridamole.

Drug	d10 (µm)	d50 (µm)	d90 (µm)
Indomethacin	23.6	75.1	265.1
Dipyridamole	31.4	76.4	211.0
