# Supplementary material for: Processing of Polyvinyl Acetate Phthalate in Hot-Melt Extrusion—Preparation of Amorphous Solid Dispersions
Source: Pharmaceutics. 2020 Apr 9;12(4):337. doi: 10.3390/pharmaceutics12040337 (PMC7238276; doi:10.3390/pharmaceutics12040337)
Supplement: Supplementary file 1 [file pharmaceutics-12-00337-s001.pdf]

pharmaceutics

Supplementary Materials: Processing of Polyvinyl Acetate Phthalate in Hot-Melt Extrusion – Preparation of Amorphous Solid Dispersions

Marius Monschke, Kevin Kayser and Karl G. Wagner *

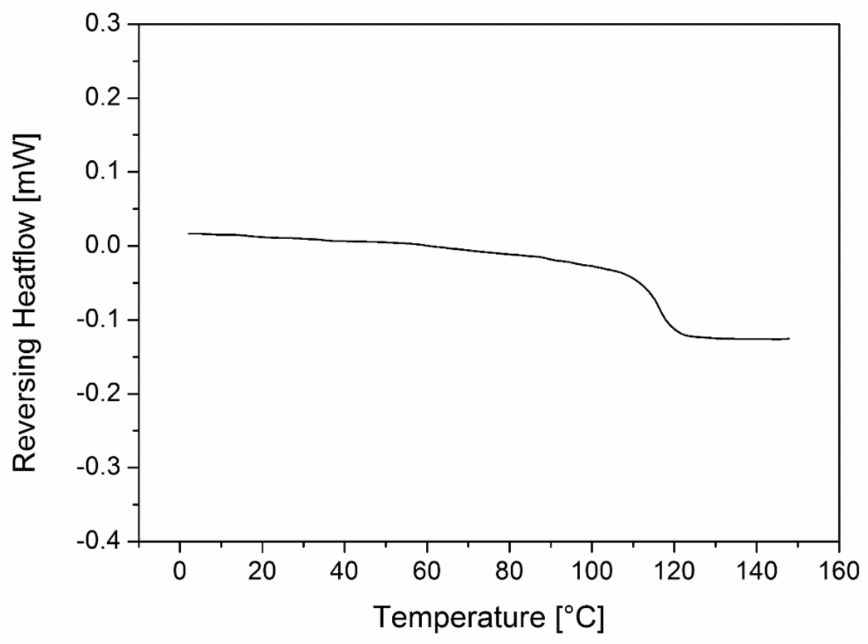

Figure S1. DSC thermogram of neat PVAP.

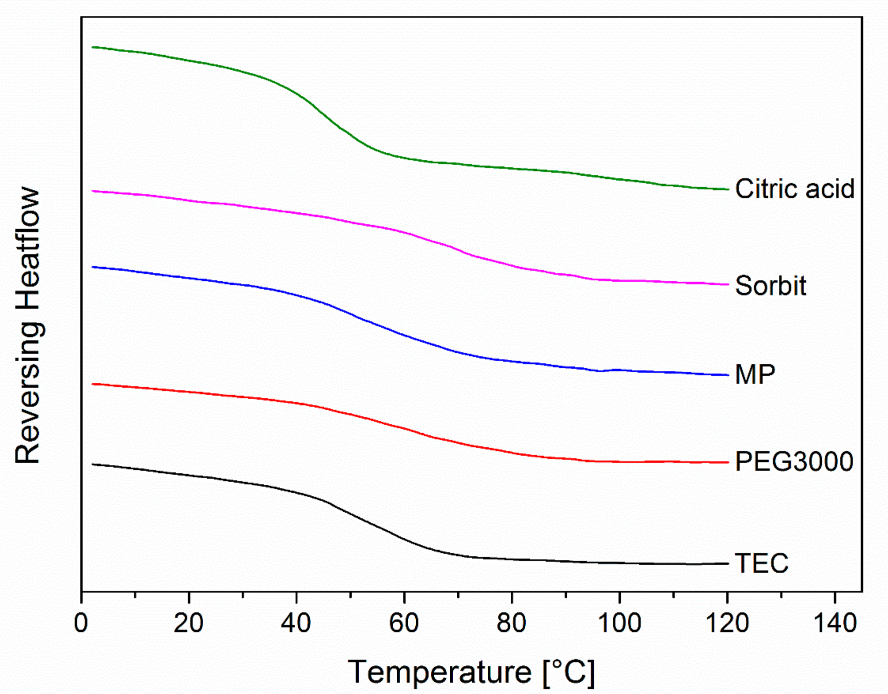

Figure S2. DSC thermograms of plasticized PVAP films.

Table S1. Compositions and glass transition temperatures of plasticized PVAP films.

Composition (90:10)	Glass Transition (°C)
PVAP:TEC	55.7 ± 0.2
PVAP:PEG3000	65.7 ± 0.1
PVAP:MP	57.2 ± 1.3
PVAP:Sorbit	69.3 ± 1.7
PVAP:Citric acid	49.6 ± 1.8

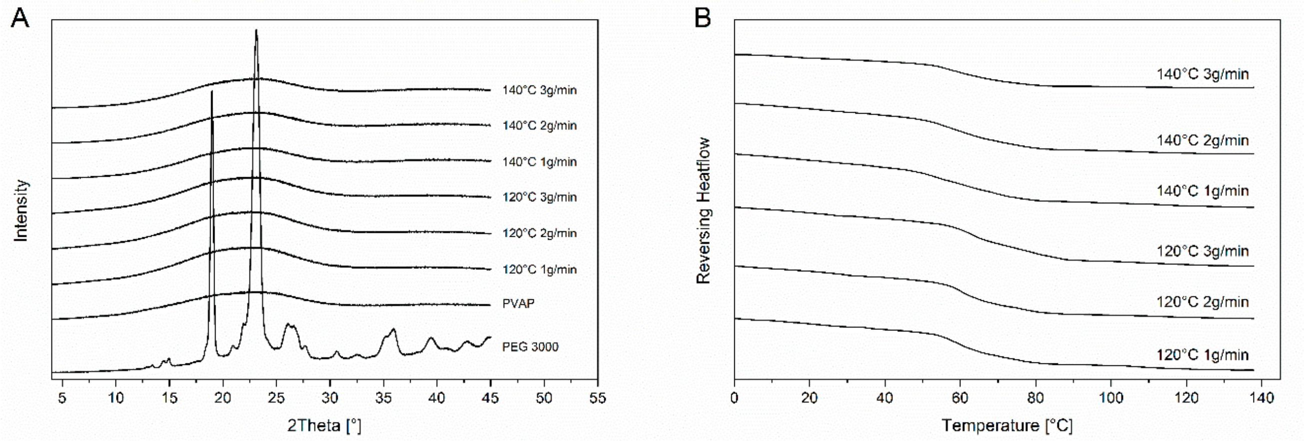

Figure S3. (A) XRPD diffractograms and (B) DSC thermograms of PVAP/PEG 3000 (90/10) mixtures extruded at various process parameters.

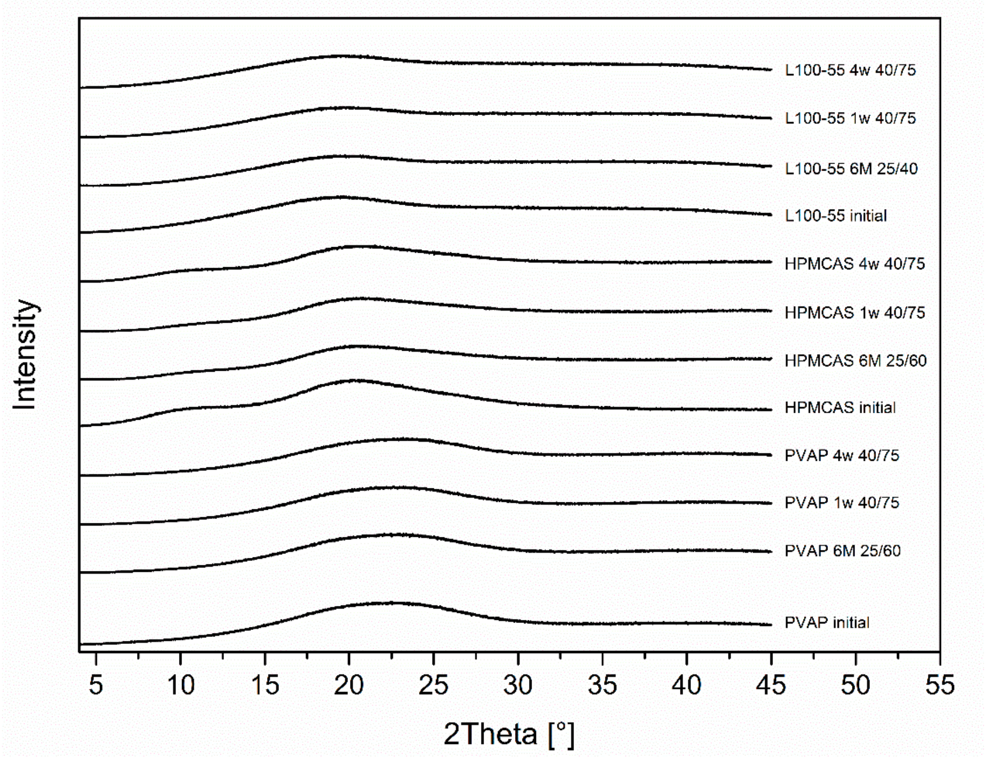

Figure S4. XRPD diffractograms of PVAP, HPMCAS and Eudragit L100-55 ASDs upon storage under different conditions.

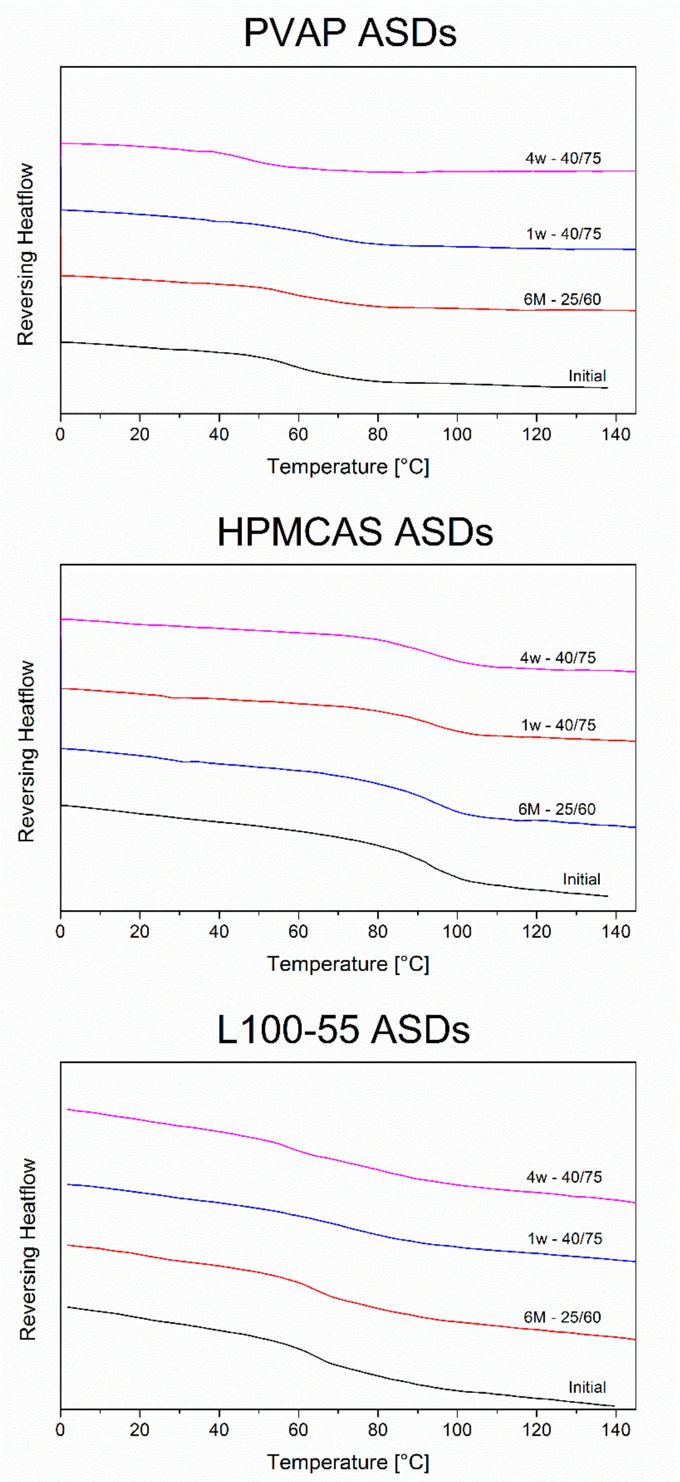

Figure S5. DSC thermograms of PVAP, HPMCAS, Eudragit L100-55 ASDs upon storage under different conditions.
